# A Case of Apparent Contact Dermatitis Caused by *Toxocara* Infection

**DOI:** 10.1155/2014/625724

**Published:** 2014-12-16

**Authors:** Rosanna Qualizza, Eleni Makrì, Laura Losappio, Cristoforo Incorvaia

**Affiliations:** ^1^Allergy Service, Istituti Clinici di Perfezionamento, 20100 Milan, Italy; ^2^Allergy/Pulmonary Rehabilitation, Istituti Clinici di Perfezionamento, 20100 Milan, Italy; ^3^General Medicine, University of Foggia, 71100 Foggia, Italy

## Abstract

Infection from *Toxocara* species may give rise to a large array of clinical symptoms, including apparent manifestations of allergy such as asthma, urticaria/angioedema, and dermatitis. We report a case, thus far not described, of contact dermatitis attributed to nickel allergy but caused by *Toxocara* infection. The patient was a 53-year-old woman presenting from 10 years a dermatitis affecting head, neck, and thorax. Patch tests initially performed gave a positive result to nickel, but avoidance of contact with nickel did not result in recovery. The patient referred to our Allergy Service in 2010 because of dermatitis to feet. Patch testing confirmed the positive result for nickel, but expanding the investigation a positive result for IgG antibodies to *Toxocara* was detected by Western blotting and ELISA. Treatment with mebendazole achieved immediate efficacy on feet dermatitis. Then, two courses of treatment with albendazole resulted in complete regression of dermatitis accompanied by development of negative ELISA and Western blotting for *Toxocara* antibodies. This report adds another misleading presentation of *Toxocara* infection as apparent contact dermatitis caused by nickel and suggests bearing in mind, in cases of contact dermatitis not responding to avoidance of the responsible hapten and to medical treatment, the possible causative role of *Toxocara*.

## 1. Introduction

*Toxocara* species is an intestinal nematode mainly affecting dogs and cats, which causes human infection when embryonated eggs excreted in dog faeces are ingested but also by eating raw or undercooked meat (from chicken, cow, pigs, rabbits, and others), the latter being a frequent mode of infection in adults. In humans, the larvae do not develop into adult worms but may migrate to various tissues and organs where they can survive for years, giving rise to a number of clinical symptoms [1–3]. Among them, apparently allergic manifestations are reported, including asthma, urticaria/angioedema, and dermatitis [4–6]. We report a case, thus far not described, of contact dermatitis diagnosed as nickel allergy but caused by* Toxocara* infection.

## 2. Case Presentation

**Figure 1:**
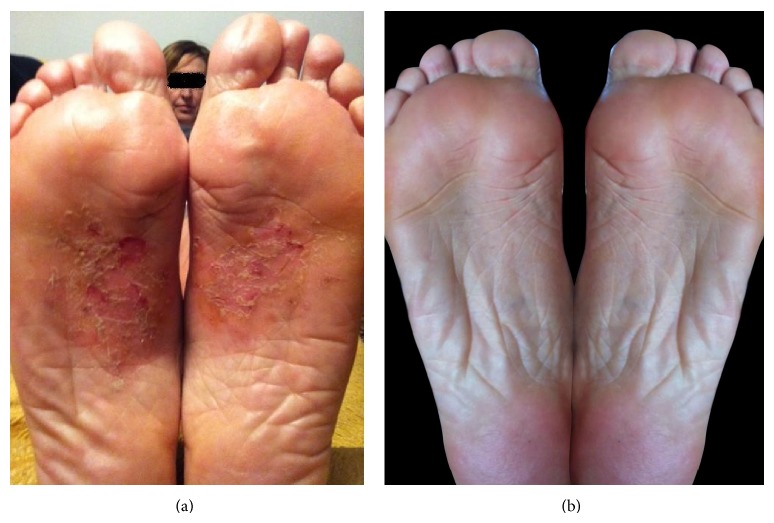
(a) Foot dermatitis before diagnosis of* Toxocara* infections. (b) Foot dermatitis after antiparasitic treatment.

**Figure 2:**
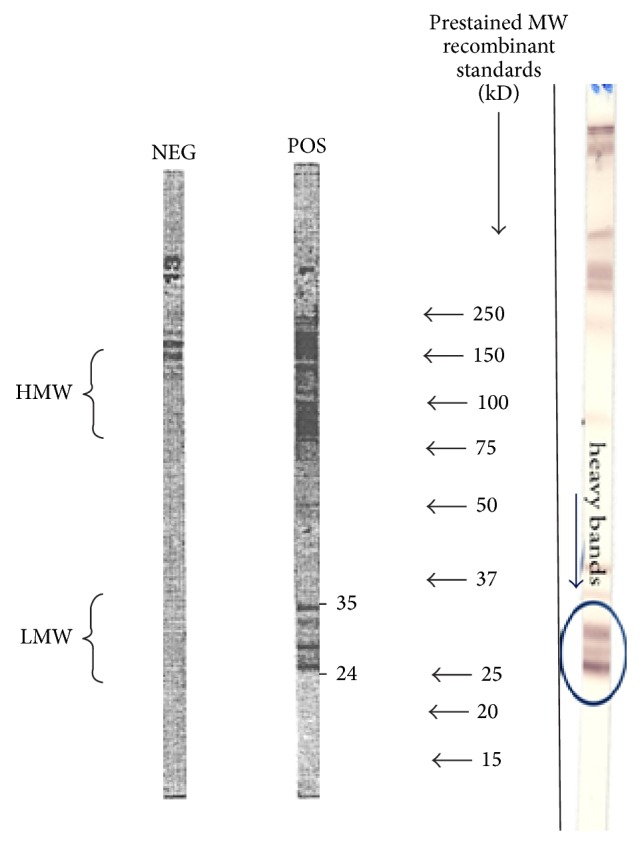
Result of Western blotting for anti-*Toxocara* IgG antibodies.

The patient was a 53-year-old woman presenting from 10 years a dermatitis affecting head, neck, and thorax. Patch tests initially performed gave a positive result only to nickel. The patient avoided any possible contact with nickel, but dermatitis recurred regularly at intervals of 6–8 months. In 2005 dermatitis also affected the sole of the right foot and was treated with topical steroids, but in the following years also edema of the foot with impaired walking occurred. The patient referred to our Allergy Service in 2010 because of the development of dermatitis also to the left foot (Figure 1(a)). Patch testing confirmed the positive result for nickel sulfate. The patients also complained about recurrent headache and asthenia especially in the morning. By routine blood tests, only peripheral eosinophilia and total IgE levels were abnormal. We required other immunological tests including ANA, ENA, and anti-*Toxocara* IgG antibodies, yielding a positive result to the latter by Western blotting (Figure 2) and ELISA using material from LTBio Diagnostics (Lyon, France).

## 3. Results and Discussion

Following the diagnosis of* Toxocara* infection, treatment with mebendazole 100 mg b.i.d. for 3 days was started, achieving immediate efficacy on feet dermatitis and edema. Other 3 courses of mebendazole treatment were performed, with dermatitis showing a mild reoccurrence, while headache and asthenia disappeared. Also peripheral eosinophilia turned to normal value. Then, two courses of treatment with albendazole 400 mg b.i.d. for 5 days were performed that were followed by complete regression of dermatitis (Figure 1(b)), accompanied by development of negative ELISA and Western blotting for* Toxocara* antibodies. This observation differs from most reports in the literature that show persistence of ELISA and Western blotting positive results for a long period of time after treatment [7].

This report adds another misleading presentation of* Toxocara* infection as apparent contact dermatitis caused by nickel. Nickel allergy is quite common, its prevalence being estimated in around 12% in a recent study [8]. This makes understandable that in a patient with dermatitis and positive response to patch test with nickel an obvious diagnosis of nickel allergy is stated. The present case shows that also this kind of clinical presentation may be sustained by an unrecognized* Toxocara* infection. Only the correct diagnosis allowed curing the 10-year long dermatitis of the patient, the causative role of* Toxocara* being supported by the immunological laboratory results. This confirms that the role of* Toxocara* infection in causing clinical manifestations of apparent allergy is often overlooked [9] and suggests bearing in mind, at least in cases of apparent contact dermatitis not responding to avoidance of the responsible hapten and to medical treatment, that the possible agent may be* Toxocara*.

## References

[1] Overgaauw P. A. (1997). Aspects of toxocara epidemiology: human toxocarosis. *Critical Reviews in Microbiology*.

[2] Despommier D. (2003). Toxocariasis: clinical aspects, epidemiology, medical ecology, and molecular aspects. *Clinical Microbiology Reviews*.

[3] Rubinsky-Elefant G., Hirata C. E., Yamamoto J. H., Ferreira M. U. (2010). Human toxocariasis: diagnosis, worldwide seroprevalences and clinical expression of the systemic and ocular forms. *Annals of Tropical Medicine and Parasitology*.

[4] Cooper P. J. (2008). Toxocara *canis* infection: an important and neglected environmental risk factor for asthma?. *Clinical and Experimental Allergy*.

[5] Gavignet B., Piarroux R., Aubin F., Millon L., Humbert P. (2008). Cutaneous manifestations of human toxocariasis. *Journal of the American Academy of Dermatology*.

[6] Qualizza R., Incorvaia C., Grande R., Makrì E., Allegra L. (2011). Seroprevalence of IgG anti-Toxocara species antibodies in a population of patients with suspected allergy. *International Journal of General Medicine*.

[7] Fillaux J., Magnaval J.-F. (2013). Laboratory diagnosis of human toxocariasis. *Veterinary Parasitology*.

[8] Mortz C. G., Bindslev-Jensen C., Andersen K. E. (2013). Nickel allergy from adolescence to adulthood in the TOACS cohort. *Contact Dermatitis*.

[9] Pinelli E., Aranzamendi C. (2012). Toxocara infection and its association with allergic manifestations. *Endocrine, Metabolic & Immune Disorders: Drug Targets*.

